# Exposure to Ambient Particulate Matter during Pregnancy: Implications for Infant Telomere Length

**DOI:** 10.1101/2023.09.17.23295692

**Published:** 2023-09-18

**Authors:** Nina E. Ahlers, Sandra J. Weiss, Jue Lin

**Affiliations:** 1 Department of Community Health Systems, University of California, San Francisco, CA, USA.; 2 Department of Biochemistry and Biophysics, University of California, San Francisco, CA, USA

**Keywords:** Air pollution, Telomere length, Prenatal exposure, Fetal programming, Pregnancy, Infants

## Abstract

**Background:**

Growing evidence suggests that air pollution may influence fetal development, with potential consequences for later health. Alteration of telomere length (TL) is one possible mediating mechanism for the link between fetal exposure to air pollution and development of disease. However, the few studies exploring associations between prenatal pollution and infant TL have assessed varied trimesters of pregnancy and shown mixed results. The aim of this study was to examine differential relationships of prenatal exposure to air pollutant PM_2.5_ during the 1^st^, 2^nd^ and 3^rd^ trimesters of pregnancy with infant TL at one month of age.

**Methods:**

Women (n=74) were recruited in obstetric clinics during their third trimester. Data on PM_2.5_ exposure for each woman’s residential area during each trimester was acquired from the regional Air Quality Management District. At one month postnatal, a salivary sample was collected from the infant which provided DNA for telomere assay. Women completed questionnaires about stressors in their lives, perceived stress, depression, and sociodemographics for inclusion as covariates. Multiple linear regression was used to analyze the aims.

**Results:**

PM_2.5_ exposure during the 2^nd^ (β =.31, p=.003) and 3rd (β =.24, p=.02) trimesters was associated with longer infant TL. Exposure in the 1^st^ trimester was not related to TL. Covariates of maternal depression and age, and infant female sex were also associated with longer TL. Variables in the model contributed to 34% of the variance in TL (F=10.58, p=.000).

**Discussion:**

Fetal programming of longer telomeres in response to pollution may have adaptive value in preparing the neonate for a postnatal environment that is less than optimal in air quality. Alternatively, longer telomeres may forecast later health risk considering established links between longer TL and diseases such as cancer. Future research needs to address how prenatal pollution interacts with TL to influence health over time.

## 1. Introduction

Air pollution is an established cause of respiratory and cardiovascular diseases, reproductive and central nervous system dysfunctions, and cancer (Dominski et al., 2021; Johnson et al., 2021; Manisalidis et al., 2020). There is growing evidence that adverse impacts of air pollution can begin as early as pregnancy (Bai et al., 2020; Proietti et al., 2013; Yan et al., 2022) and have long term impacts in later life (Iglesias-Vázquez et al., 2022; Lubczyńska et al., 2021). These findings reinforce the validity of a widely recognized theory, the Developmental Origins of Health and Disease (DOHaD), which proposes that in utero exposures program fetal development to influence health outcomes throughout life (Lacagnina, 2019; Swanson et al., 2009). Fetal exposure to air pollutants can occur through cross-placental transfer of particulate matter or metabolized molecules of air pollutants (Bové et al., 2019; Kim et al., 2018; Liu et al., 2020).

Shortening of telomere length (TL) is one possible mediating mechanism for the link between fetal exposure to air pollution and later development of disease (Hermanova et al., 2022). Telomeres are protective caps at the end of chromosomes, which reduce their risk of erosion (Blackburn et al., 2015). A recent systematic review and meta-analysis indicated that 94.8% of participants from 19 studies experienced shorter TL from exposure to air pollution (Zhao et al., 2018). However, few studies have examined the association between air pollution and TL in utero or among infants.

Research suggests that the integrity of telomeres can affect the way in which DNA unfolds, with early life programming of an individual’s telomere biology accounting for a large proportion of later health and disease (Bosquet Enlow et el, 2019; Entringer et al., 2018; Shalev et al, 2013). Programming of adult TL may begin as early as embryogenesis (2004) and is strongly predicted by newborn TL (Entringer et al., 2018). However, newborn telomere length appears to be highly variable (Factor-Litvak et. al., 2016; Okuda et. al., 2002), implicating the prenatal stage as a critical window of vulnerability to early life exposures.

Particulate matter with aerodynamic diameter ≤ 2.5 μm (PM_2.5_) has been the primary type of pollution studied during pregnancy. One study examining the 1st trimester of pregnancy found that increased PM_2.5_ was associated with shorter TL in cord blood of newborns (Rosa et al., 2019). Three studies of the 2^nd^ trimester reported mixed results, with two finding that higher PM_2.5_ exposure was related to shorter TL in cord blood of newborns (Lee et al., 2020; Martens et al., 2017) while a 3^rd^ study found that higher PM_2.5_ levels during the 2^nd^ trimester were associated with longer TL (Rosa et al., 2019). For 3^rd^ trimester PM_2.5_ exposure, three studies also reported mixed findings from cord blood, with two noting that higher pollution predicted shorter TL (Scholten et al., 2021; Song et al., 2019) and a 3^rd^ study finding pollution was associated with longer TL (Rosa et al., 2019). Clearly, research to date indicates no consensus regarding the nature of the relationships between air pollution during various trimesters of pregnancy and infant TL.

The purpose of this study was to advance existing knowledge of the effects of pollution exposure during different trimesters on infant TL. Our specific aim was to assess the differential relationships of prenatal exposure to air pollutant PM_2.5_ during the 1^st^, 2^nd^ and 3^rd^ trimesters of pregnancy on infant telomere length at one month of age.

## 2. Materials & Methods

### 2.1 Sample and Procedures

Our sample included pregnant women (n=74) enrolled in a longitudinal cohort study funded by the National Institutes of Health and the data collection procedures were previously detailed ((Ahlers and Weiss, 2021). In brief, English and Spanish speaking women from obstetric clinics affiliated with a large University medical center were eligible to participate if they were ≥18 years old and ≥28 week’s gestation between 2015 and 2019. Based on requirements of the larger cohort study, exclusion criteria included ongoing steroid use or a history of endocrine conditions, smoking, serious medical problems or cognitive impairment.

Archived data and sample specimens were accessed January 5^th^, 2023, for the purpose of this study. The information we accessed could identify individual participants during and after data collection. We acquired data on particulate matter (PM_2.5_) within each woman’s residential area from public records of the air quality control district in the region, identifying levels present during each trimester of the woman’s pregnancy. Salivary samples for telomere analysis were collected from the infant at one month of age. Women also completed measures of their demographics, perceived stress, the stressors they experienced, and their depression as potential covariates. All participants provided written consent and the study was approved by the University of California of San Fransico’s (UCSF) Institutional Review Board for Human Research Protection before the study began.

### 2.2 Air Pollutant (PM_2.5_) Exposure During Pregnancy

We chose particulate matter with a diameter of 2.5 micrometers (PM_2.5_) or less as the air pollutant to measure because of its association to TL outcomes and hormonal dysregulation in previous research (Ahlers and Weiss, 2021; Zhao et al., 2018). Pregnancy air pollution measures were based on data from the Bay Area Air Quality Management District (BAAQMD). They gather 24-h average PM_2.5_ data from 33 air monitoring stations across 9 counties, with fixed locations based on knowledge of population density and local wind patterns. BAAQMD sites were determined after analyzing preliminary air quality measurements collected from field studies, temporary monitoring studies, and mobile monitoring data (Knoderer et al., 2019). Our participants resided within 4 BAAQMD counties and data from 6 monitors were used. PM_2.5_ measures were estimated for each participant according to the nearest monitoring station to their residence at time of enrollment. Maternal residence (defined as the primary residence of the entire prenatal period) was geocoded with Google Earth. This approach to measurement is supported by Kim et al. (2005) who suggest that central fixed-site measurements of PM_2.5_ can be treated as a proxy measure for personal exposure to PM_2.5_ within a 15.5 mile radius. The average distance between maternal residence and the nearest PM_2.5_ monitoring station in our study was 2.39 miles and ranged between 0.28 to 5.63 miles. We calculated four air pollution measures for the analyses, including the woman’s average PM_2.5_ exposure across all trimesters and estimates for each clinically defined trimester (1st trimester: 1–13 weeks, 2nd trimester: 14–27 weeks, 3rd trimester: 28 weeks-delivery).

### 2.3 Infant Telomere Length

Saliva for DNA was collected from infants at approximately one month of age using an Oragene DNA Collection Kit (DNA Genotek, Inc). The kit contained 5 small sponges which were each inserted sequentially for one minute in the infant’s mouth. Each sponge was moved around the upper and lower cheek pouches for approximately 30 seconds on each side of the mouth, and then placed in a collection tube with stabilizing solution at room temperature until assayed for telomere length. Research has validated saliva samples as a high-quality source of DNA for genomic applications that is equivalent to DNA from blood for downstream applications (Hu et al., 2012; Tomiyama et al., 2012; Van den Bergh et al., 2017). Salivary telomere length correlates with leukocyte telomere length and known correlates of telomere length (e.g., age and adversity) as measured by blood leukocytes (von Zglinicki, 2002; Wilkgren et al., 2012; Zhao et al., 2018).

Genomic DNA was purified from 500 μl of saliva with the DNA Agencourt DNAdvance kit (cat# A48705, Beckman Coulter Genomics Inc. Brea CA). DNA was quantified by measuring OD260 with a NanoDrop 2000c Spectrophotometer (Nanodrop Products, Wilmington, DE, USA) and ran on 0.8% agarose gels to check the integrity. Samples that passed the quality control of OD260/OD280 between 1.7-2.0, concentration greater than 10ng/ μl and no degradation were used for telomere length measurement. All except one sample passed quality control.

Relative telomere length was measured by quantitative polymerase chain reaction (qPCR), expressed as the ratio of telomere to single-copy gene abundance (T/S ratio) (Cawthorn et al., 2002; Lin et al., 2010). The telomere qPCR primers were tel1b [5'-CGGTTT(GTTTGG)5GTT-3'], used at a final concentration of 100 nM, and tel2b [5'-GGCTTG(CCTTAC)5CCT-3'], used at a final concentration of 900 nM. The single-copy gene (human beta-globin) qPCR primers were hbg1 [5'-GCTTCTGACACAACTGTGTTCACTAGC-3'], used at a final concentration of 300 nM, and hbg2 [5'-CACCAACTTCATCCACGTTCACC-3'], used at a final concentration of 700 nM. The final reaction mix consisted of the following: 20 mM Tris-hydrochloride, pH 8.4; 50 mM potassium chloride; 200 μM each deoxyribonucleotide triphosphate; 1% dimethyl sulfoxide; 0.4x SYBR green I; 22 ng Escherichia coli DNA; 0.4 Units of platinum Taq DNA polymerase (Invitrogen Inc., Carlsbad, CA), and approximately 6.6 ng of genomic DNA per 11 microliter reaction. A 3-fold serial dilution of a commercial human genomic DNA (Sigma-Aldrich, cat#11691112001) containing 26, 8.75, 2.9, 0.97, 0.324 and 0.108ng of DNA was included in each PCR run as the reference standard. The quantity of targeted templates in each sample was determined relative to the reference DNA sample by the maximum second derivative method in the Roche LC480 program. The reaction was carried out in a Roche LightCycler 480 in 384-well plates, with triplicate wells for each sample. Dixon Q test was used to exclude outliners from the triplicates. The average of the T and S triplicate wells after outliner removal was used to calculate the T/S ratio for each sample.

The telomere (T) thermal profile consisting of denaturing at 96°C for 1 minute followed by 30 cycles of denaturing at 96°C for 1 second and annealing or extension at 54°C for 60 seconds with fluorescence data collection. The single copy gene (S) thermal profile consisting of denaturing at 96°C for 1 minute followed by 8 cycles of denaturing at 95°C for 15 seconds, annealing at 58°C for 1 second, and extension at 72°C for 20 seconds, followed by 35 cycles of denaturing at 96°C for 1 second, annealing at 58°C for 1 second, extension at 72°C for 20 seconds, and holding at 83°C for 5 seconds with data collection. The T/S ratio for each sample was measured in duplicate runs, each with triplicate wells. When the duplicate T/S values disagreed by more than 7%, the sample was run a third sometimes. The two closest values were used. 23% of samples were run a third time. The inter assay coefficient of variation (CV) for this study is 2.3%±1.6%. Intra-class correlation of duplicate DNA extraction was not estimated for this specific study but was reported to be 0.95 (CI: 0.911-0.972) from another study using saliva samples collected in the same kit, the same DNA extraction kit and the same telomere length assay (Smith et al., 2022). Detailed protocol can be found on the Telomere Research Network’s website (https://trn.tulane.edu/wp-content/uploads/sites/445/2021/07/Lin-qPCR-protocol-01072020.pdf).

### 2.4 Covariates

Data were collected on 9 covariates to control for their effects if needed in testing of the aims: maternal age, educational level, income, perceived stress, exposure to stressors and depression as well as infant sex, gestational age and birth weight. Infant sex, gestational age, and birth weight were identified from medical records while maternal demographic data was acquired from a sociodemographic questionnaire. The three other covariates (stressors, stress and depression) were measured with standardized questionnaires. Exposure to stressors was measured by *The Crisis in Family Systems (CRISYS) Questionnaire* (Berry et al. 2001; Berry et al., 2006). Respondents were asked to identify whether they had experienced any of 64 major life events in the past 6 months within 11 domains: financial, legal, career, relationship, home safety, neighborhood safety, medical issues (self and others), home, prejudice, and authority. The number of adverse life events was identified for each woman, with higher scores indicating greater exposure to stressors. Maternal perceived stress was evaluated using the *Perceived Stress Scale* (PSS; Cohen et al, 1983). The PSS measured the degree to which women felt that their lives were unpredictable, uncontrollable, and overloaded with stressors within the last month. Each of 10 items was rated on a 5-point Likert scale, with a total higher score indicating greater perceived stress. Maternal depression was assessed with the 9 item *Patient Health Questionnaire* (PHQ-9). Respondents rated how frequently they had experienced depressive symptoms over the past two weeks on a scale ranging from “not at all” to “nearly every day.” Total scores range from 0 to 27 points, with diagnostic ranges that include minimal (1-4), mild (5-9), moderate (10-14), moderately severe (15-19), and severe (20+) symptoms of depression. A score of >10 has been demonstrated to have both a sensitivity and specificity of 88% for a diagnosis of major depression (Kroenke et al., 2002).

### 2.5 Analysis

Descriptive statistics were calculated to characterize the sample. Variables were examined for linearity and normality; transformations were performed where needed to achieve normal distributions. Stepwise multiple linear regression procedures were used to examine the association between air pollution exposure during pregnancy and infant telomere length, including which covariates to retain in the final models. Two separate models were tested: one for average PM_2.5_ exposure across all trimesters and a second for the specific effects of PM_2.5_ exposure during each of the trimesters. Covariates were entered into the model in stepwise fashion. At each step, the covariate not in the equation that had the smallest probability was entered. Variables already in the regression equation were removed if their probability became sufficiently large. Entry terminated when no more variables were eligible for inclusion or removal. The best fit models were identified using the log likelihood-ratio test application. Analyses were conducted using Stata version 16 (StataCorp, College Station, TX).

## 3 Results

### 3.1 Sample Characteristics

Sample characteristics and distributions for key covariates are displayed in Table 1. 74 women were included in the study (M=33 yrs), with ~41% reporting an annual household income of $51,000 or less (32% reported $21,000 or less). About 55% were from diverse racial and ethnic backgrounds, including 20% who identified as Black/African American and 20% as Hispanic/Latina. The average prenatal PM_2.5_ exposure was 8.8μg/m^3^, with exposure estimates ranging between 5.5 and 14.4μg/m^3^ across pregnancy. Infant telomere T/S ratios had a mean value of 2.71 and ranged between 2.07 to 3.63.

### 3.2 Relationships of Prenatal PM_2.5_ Exposures to Infant Telomere Length

Table 2 shows a significant effect of average PM_2.5_ exposure across pregnancy on infant TL length (β =0.466, p=0.000). According to our findings, per every 1 ug/m3 increase of PM_2.5_ exposure across pregnancy, infant TL increased by 0.466 log units (p<0.000). Maternal depressive symptoms (β =0.225, p=0.025) and maternal age (β =0.218, p=0.032) had significant positive associations to TL. In addition, male infants had significantly shorter TL than female infants (β =−0.258, p=0.010). The variables in the model contributed to 34% of the variance in TL (F=10.58, p=0.000). All covariates were included in the preliminary model testing, but only the final variables were retained due to their significant effects on TL and goodness of fit based on the likelihood-ratio test. Preliminary bivariate correlation coefficients for all variables in the study are presented in supplemental Table 1S.

Table 3 presents differential effects for each of the trimester PM_2.5_ exposures, adjusted for significant covariates. First trimester exposure to PM_2.5_ was not significantly related to infant TL in either bivariate correlations or after adjusting for covariates. Second and third trimester exposure to PM_2.5_ had significant positive associations to TL (β =0.310, p=.003; β =0.241 p=0.021; respectively). Like effects shown in Table 2, prenatal depressive symptoms, maternal age, and infant female sex were positively associated with longer infant TL.

## 4 Discussion

Results of this study indicate that fetal exposure to particulate matter in the air (PM_2.5)_ during pregnancy is significantly associated with longer infant TL at one month of age. The strongest effect of PM_2.5_ was found for overall pregnancy exposure. However, results suggest that exposure during the 2^nd^ trimester may account for the greatest contribution to infant TL, followed by exposure during the 3^rd^ trimester. Although average PM_2.5_ exposure was highest during the 1^st^ trimester, air pollution during this trimester was not associated with TL. These differential findings among trimesters suggest that the first trimester may not be the time of greatest fetal vulnerability to particulate matter. In addition, based on our results and previous findings, the amount of PM_2.5_ exposure does not appear to provide a primary explanation for variation in TL (Lee et al., 2020; Roa et al., 2019; Song et al., 2019).

### 4.1 Exposure to PM_2.5_ During Pregnancy and Infant Telomere Length

Women in our study experienced an average PM_2.5_ exposure level of 8.8 μg/m^3^ throughout their pregnancy. Although this exposure level is actually below the standardized safety limit of 12.0 μg/m^3^ (National Ambient Air Quality Standards, 2020), it was still associated with telomere length. However, it is important to note that concentration levels peaked as high as 23 μg/m^3^, 21 μg/m^3^, and 18 μg/m^3^ during each progressive trimester, respectively. Our findings are consistent with other studies that have identified the 2^nd^ and 3^rd^ trimesters of pregnancy as critical windows of vulnerability to PM_2.5_ exposure. Rosa et. al. (2019) also found significant associations between increased PM_2.5_ exposure during the 2^nd^ and 3^rd^ trimesters and elongated infant TL. Scholten et al. (2021) found a positive association between 2^nd^ trimester exposure and infant TL but a negative association with exposure during the 3^rd^ trimester.

Despite this previous support for our findings, there are some notable differences between our study and all previous studies examining these relationships. Other research examined TL in cord blood cells collected from the infant after delivery (Lee et al., 2020; Marten et al., 2017; Rosa et al., 2019; Song et al., 2019; Scholten et al., 2021) in contrast to our study which analyzed infant salivary samples during the first month of life. Thus, comparison is difficult because of the differing developmental timepoints and the use of different tissue sources. While most studies have found shorter telomeres in blood from the effect of air pollution on adults (Zhao et al., 2019), one study examining salivary TL among children 8-9 years of age found that greater PM_2.5_ exposure was associated with longer TL (Walton et al., 2016). In addition, 3 of the 5 studies examining the effect of prenatal exposure to particulate matter on cord blood TL among newborns reported elongated telomeres. Although mixed findings do exist, our study adds to growing evidence supporting the hypothesis that air pollution exposure during pregnancy may lead to longer TL during early life.

Maternal inhaled particulate matter can directly cross the placental barrier into the placenta or fetal circulation (Wick et al., 2010), resulting in the production of DNA damaging reactive oxygen species (ROS) from the surface of the particles (Blackburn et al., 2015, Kawanishi et. At., 2004) and activation of inflammation in cells (Risom et al., 2005). Both processes may cause mutations and shortening of telomeres. However, inflammation-inducing particulate matter may not always cross through the placenta (Hawkins et al., 2018) but instead stimulate the placenta in ways that result in low grade inflammatory fetal reactions (van Den Hooven et al., 2012). These low grade inflammatory reactions have been associated with elongated telomeres (Dioni et al., 2011; Hodes et al., 2002; Weng et al., 1997). As noted by Scholten et al. (2021), alterations caused by low grade systemic inflammation may reflect both expansion of cellular subpopulations with longer telomeres (T and B lymphocytes), as those proliferate faster, and a greater number of leukocytes from the bone marrow which typically produces longer telomeres due to their immaturity (Hodes et al., 2002; Thiede et al., 2000). Up-regulation of telomerase (an enzyme that extends telomeres by generating DNA) may also be involved as a mechanism to counteract erosive effects of inflammation or air pollutants on telomeres (Vasu et al., 2017; Wang et al., 2020). The roles of both inflammation and telomerase in maintenance and elongation of fetal telomeres need further exploration.

Some early alterations in biological systems, such as the telomere system, may have adaptive value in preparing the infant for potential adversities in the environment after birth (Gluckman and Hanson, 2004). It has been suggested that mild to moderate levels of early life adversity may have positive, adaptive effects on fetal programming by creating longer telomere length at birth (Bosquet Enlow M et al., 2021; Rideout, 2022). In this way, longer telomeres could provide a compensatory and protective foundation for postnatal health when potentially adverse air quality is expected.

Alternatively, it is important to note that longer telomeres are not always indicative of a protective health status or advantageous outcomes. For example, multiple studies have reported that exposure to carcinogens is associated with longer telomeres (Ameer et al., 2016; Bassig et al., 2014; Mitro et al., 2016; Shin et al., 2010; Villarreal et al., 2019) and that longer telomeres are a risk factor for many types of cancer (Haycock et al., 2017; Zhang et al., 2017). With this view in mind, exposure to particulate matter in utero may place the infant at future risk for later health problems. The developmental trajectory of health outcomes related to the interaction between prenatal pollution exposure and TL will be essential to study.

### 4.2 Associations of Covariates with Infant Telomere Length

Although not a focus of our research aims, results indicate that increased prenatal depressive symptoms, older maternal age, and infant female sex were also associated with longer infant telomeres. Few studies to date have examined the relationship between prenatal depression and infant TL, with mixed findings. Ammala et al. (2020) and Wojcicki et al (2015) found no relationship between prenatal maternal depression and infant TL. In other studies, prenatal depressive symptoms have been associated with shorter cord blood TL among male infants (Bosquet et al., 2018) and shorter placental TL in female infants (Garcia-Martin et al., 2021). In a final study, prenatal depression was associated with longer TL among males only (Bosquet et. al., (2021).

While paternal age is a well-established predictor of newborn TL (Eisenber and Kuzawa, 2018), maternal age has shown no significant effects on infant TL (e.g., Chen et al., 2022; Lee et al., 2017). We found one study reporting an association between maternal age at birth and longer infant TL (Verner et al., 2021).

Our findings regarding infant sex are consistent with other studies reporting shorter telomeres among males versus females in the association to increased prenatal PM_2.5_ exposure (Lee et al., 2020; Song et al., 2019) and other environmental exposures (Herlin et al., 2019). However, one previous study found that prenatal PM_2.5_ exposure was associated with shorter TL for female infants than males (Rosa et al., 2019) and another reported no sex differences (Martens et al., 2017). Sex differences in telomere dynamics may be associated with sex-related hormonal conditions during intrauterine life (Aviv, 2002) but these conditions have not been carefully studied. More research is clearly needed to understand how maternal depression, maternal age and infant sex may influence infant TL and interact with air pollution in determining effects on telomere length.

### 4.3 Strengths and Limitations

The strengths of our study include a diverse sample, the high temporal resolution of the PM_2.5_ estimates, examination of pollution across all trimesters, and adjustment for salient covariates. Given our exposure estimates are extracted from the nearest single site monitor from a network of ground monitors, a limitation of our study is that we do not account for further personalized exposure estimates for each participant from the workplace or indoor household exposure levels that can affect exposure variation. We also didn’t include other air pollutants in our analysis that can potentially modify or confound our observed effect associations. Lastly, our ability to understand the long term effects is limited by the cross-sectional design of our study.

### 4.4 Conclusions

Our findings indicate that fetal exposure to greater air pollution during pregnancy is related to longer telomeres among infants at 1 month of age. Fetal programming of longer telomeres in response to pollution may have adaptive value in preparing the neonate for a postnatal environment that may be less than optimal in air quality. Alternatively, longer telomeres may not be indicative of protected health status, considering what is known about the link between longer telomeres and cancer risk. In this context, elongated telomeres could place the neonate at future risk for later health problems. It will be essential to better understand mechanisms responsible for pollution-related programming effects on TL, such as telomerase up-regulation, inflammation, oxidative stress, or epigenetic alterations. Future research also needs to address how prenatal pollution exposure interacts with telomere length to influence health outcomes over time.

## Figures and Tables

**Table 1.** Sample Characteristics and Covariates

Variable	Unit or Category	Mean (Range) or N (%)
**Maternal age**	Years	33 (21-44)
**Maternal education**		
	Elementary school	3 (4%)
	High school or GED	15 (20%)
	Some college	15 (20%)
	Bachelor’s degree	16 (22%)
	Master’s degree	10 (14%)
	Advanced degree	15 (20%)
**Household income**		
	Less than $15,000	14 (19%)
	$15,000-$30,999	14 (19%)
	$31,000-$50,999	3 (4%)
	$51,000-$100,999	2 (3%)
	$101,000-$149,999	10 (13%)
	$150,000+	31 (42%)
**Stressors**	Number of Events	6.8 (0-39)
**Perceived stress**	Continuous Score	14.6 (3-31)
**Maternal depression**	Continuous Score	5.8 (0-18)
**Infant sex**		
	Male	38 (51%)
	Female	36 (49%)
**Infant gestational age**	Weeks	36.4 (27.3-41.6)
**Infant birth weight**	Grams	2771.6 (700-4650)
**Infant telomere length**	T/S ratios*	2.71 (2.07-3.63)
**PM_2.5_ exposure**		
**1^st^ trimester exposure**	μg/m^3^	9.4 (4.2-22.7)
**2^nd^ trimester exposure**	μg/m^3^	8.8 (3.9-21.1)
**3^rd^ trimester exposure**	μg/m^3^	8.3 (4.2-19.7)
**Average pregnancy exposure**	μg/m^3^	8.8 (5.5-14.4)

* Telomere length ratio values were log transformed.

**Table 2:** Regression Model for Effects of Average Prenatal PM_2.5_ Exposure on Infant Telomere Length^a^

	Effects on Infant Telomere Length		
Model Covariates	β (95% CI)	Standard Error	P-value
Average Prenatal PM_2.5_	0.466 (0.275 – 0.657)	0.096	0.000
Depressive Symptoms	0.225 (0.029 - 0.421)	0.098	0.025
Maternal Age	0.218 (0.020 - 0.417)	0.099	0.032
Infant Male Sex	−0.258 (−0.451 – −0.065)	0.097	0.010

a R-adj=0.34.

**Table 3.** Regression Model for Effects of Prenatal PM_2.5_ Exposure in Each Trimester on Infant Telomere Length^a^

	Effects on Infant Telomere Length		
Model covariates	β (95% CI)	Standard Error	P-value
T1 PM_2.5_	0.184 (−0.015 – 0.382)	0.099	0.069
T2 PM_2.5_	0.310 (0.110 – 0.509)	0.099	0.003
T3 PM_2.5_	0.241 (0.038 – 0.443)	0.101	0.021
Depressive Symptoms	0.207 (0.004 - 0.410)	0.102	0.046
Maternal Age	0.211 (−0.011 – 0.412)	0.101	0.039
Infant Male Sex	−0.245 (−0.442 – −0.049)	0.098	0.015

a R-adj=0.20.
